# A Primary Care Approach to Constipation in Adults with Intellectual and Developmental Disabilities

**DOI:** 10.1155/2021/3248052

**Published:** 2021-11-15

**Authors:** Reshmi Mathew, Barrett O. Attarha, Govind Kallumkal, Morgan Cribbin, Christopher Izzo, Linda Edwards, Rafik Jacob

**Affiliations:** ^1^University of Florida College of Medicine, Jacksonville, USA; ^2^University of Florida College of Medicine, Gainesville, USA

## Abstract

Constipation is a condition that is very prevalent and is reported in up to 40 percent of individuals with intellectual and developmental disabilities (IDD). Constipation in this patient population is most commonly secondary to neuromuscular abnormalities, immobility, suboptimal diet, and medication side effects. History taking is frequently limited in adults with IDD due to communication barriers, often leading to a missed diagnosis of constipation. Inadequately treated constipation may lead to adverse effects including behavioral disturbances, fecal impaction, intestinal obstruction, and even death from intestinal perforation and sepsis. As a result, a high index of suspicion must exist for this patient population. Treatment in these patients requires an individualized approach, to reduce the constipation and its associated health complications.

## 1. Introduction and Background

IDD is characterized by significant limitations in intellectual functioning and adaptive behavior as expressed in conceptual, social, and practical adaptive skills and manifested before the individual attains age 22 [1]. IDD can begin at any point during a person's developmental period, most often before a person is born, and generally lasts for the duration of that person's life [2]. The ever-increasing population of adults with IDD and their increasing life expectancy due to improved medical care demand an evolution in their care. Primary care physicians (PCPs) who are assuming care of this patient population should be aware of the complexities of their illnesses [3]. It was concluded that the lack of medical knowledge of the PCPs may have contributed to increased morbidity and mortality of adults with IDD living in communities [4]. This led the American Academy on Developmental Medicine and Dentistry to issue a health disparity consensus statement, asserting that the majority of physicians and dentists have limited knowledge regarding the health and psychosocial needs of this population primarily due to a lack of exposure and training.

It is well established that adults with IDD are more frequently affected throughout their life by certain chronic diseases more than their peers without disabilities. These conditions include obesity, seizure disorders, cardiovascular disease, thyroid disease, and constipation [5].

Constipation is a syndrome defined by bowel symptoms of difficult or infrequent passage of stool, hardness of stool or feeling of incomplete evacuation. The Rome III criteria separate constipation into two syndromes—functional constipation and constipation-predominant irritable bowel syndrome. Constipation can be classified as primary constipation which is idiopathic or functional and secondary constipation which is usually secondary to medical conditions or medications [6].

A recent systematic review on the prevalence of constipation in people with IDD identified 31 relevant studies, of which 14 reported constipation rates of 50% or more and 21 reported rates over 33%, and over 25% of people with IDD received a repeat prescription for laxatives in one year, compared to 0.1% of people without IDD [7]. In a further recent study of 99 people with severe or profound intellectual and motor disabilities, 94% had constipation [8]. Constipation has been linked to discomfort, pain, anxiety, and behavioral difficulties in those patients [9]. These patients are at increased risk of constipation and its complications due to several reasons, among which are neuromuscular abnormalities, immobility, suboptimal diet, and medication side effects. The purpose of this article is to increase the awareness of this common problem among primary care providers who are not familiar with this specific patient population, reviewing the etiology, current approaches for the diagnosis, prevention, and treatment. This increased awareness will have a positive impact on functioning and wellbeing of adults with IDD.

## 2. Review

### 2.1. Etiology of Constipation in Adults with IDD

Several factors put adults with IDD at increased risk of constipation. Constipation prevalence among adults with a higher level of IDD (IQ < 50), living in group homes, ranged from 26 to 60% and is correlated with being nonambulant, neurologic impairment, and use of psychotropic medications [10].

Patients with IDD often have difficulties in eating a balanced diet [4]. These patients are less likely to consume foods that support healthier bowel habits, such as a diet rich in whole grains, vegetables, and fruits [11]. Frequently, patients with IDD have food preferences that are not consistent with a fiber-rich diet.

In addition, these patients often rely on their caretakers for much of their nutrition. Good knowledge about nutrition among caretakers is important, as they have a primary role in food provision for these patients. This can especially be true as caregivers may reward good behavior with less than ideal food choices. One study showed that caretakers had a significantly lower level of knowledge about nutrition than the general population [8]. Therefore, patients with IDD are often provided with and consume less optimal diets for regular bowel movements [12]. Promotion of healthy dietary and lifestyle modifications in patients with IDD will require involvement from both healthcare providers and caregivers [9].

Patients with IDD are also less likely to engage in physical activity due to their disability [9], further contributing to constipation. The Physical Activity Guidelines for Americans recommend that patients with IDD attempt to meet the same physical activity recommendations as healthy adults [13].

Approximately 1/3 of patients with IDD present with emotional dysregulation and challenging behaviors [13]. Various antipsychotic and antimuscarinic medications are used to manage these conditions [14]. The antimuscarinic effects of these medications cause decreased colonic motility and subsequent constipation [15]. Patients using antipsychotic medications are 1.9 times more likely to experience constipation than the general population [16]. Progressions from constipation to ileus, bowel obstruction, bowel ischemia, and even death are not uncommon complications in patients prescribed clozapine [17].

Conditions such as hypothyroidism and panhypopituitarism may also contribute to increased constipation in this patient population. Hypothyroidism, for instance, is more prevalent and occurs at an earlier age in patients with Down syndrome, accounting for 25.3 to 60% of cases [18]. 70% of adults with quadriplegic cerebral palsy (CP) are commonly presented with constipation. In patients with quadriplegic CP, the origin of constipation is organic and is secondary to extraintestinal abnormalities. Dryness of the stools is a result of inadequate water and food intake due to dysphagia and slow peristalsis due to rigid abdominal muscles and frequent use of medications like antiepileptics and muscle relaxants [19, 20].

### 2.2. Diagnosis

A thorough history and physical exam can usually rule out most causes of constipation [21]. However, a thorough history may be difficult to obtain in patients with IDD. For patients with limited communication abilities, the medical history should be provided by the caregiver or family member accompanying the patient. A comprehensive physical examination should include an abdominal examination, visual perianal examination, and digital rectal examination [22]. The provider should begin with a careful perianal visual examination. The visual inspection will allow for the identification of anal fissures, anal fistulas, and external hemorrhoids. A rectal examination is often the most helpful component of the physical exam in the evaluation of constipation. A digital rectal examination can provide useful information on fecal impaction or rectal masses [22].

Identification of underlying metabolic disorders is also important in the assessment of constipation [22]. Routine laboratory tests that are helpful in the evaluation of constipation include thyroid function tests, serum calcium, glucose, electrolytes, complete blood count, and urinalysis [21]. Patients with alarm features including blood in stool, weight loss, anemia, and abdominal or rectal masses should undergo colonoscopy to rule out colon cancer. Endoscopy should also be considered in patients who experience constipation refractory to medical management and in patients aged 50 years and older who have not undergone age-appropriate colon cancer screening [6]. A plain radiograph of the abdomen is relatively inexpensive and frequently used in the diagnosis of constipation [23]. However, recent studies have shown limited value in obtaining a plain radiograph due to interobserver variation in the assessment of stool burden and poor correlation with colonic transit times [24].

Physiologic testing is only required in patients with refractory symptoms who do not respond to conventional treatment methods [21], such as constipation refractory to dietary fiber supplementation and/or over-the-counter laxatives [6]. If there are suspicions for a defecatory disorder, anorectal manometry and balloon expulsion are the initial physiologic tests of choice [21]. Defecography, although very difficult to perform in this patient population, may be considered if the above tests are equivocal or if there are suspicions of a structural abnormality in the rectum that is contributing to constipation, such as for patients suspected of having rectal prolapse [22].

### 2.3. Common Complications of Constipation in Adults with IDD

**Figure 1:**
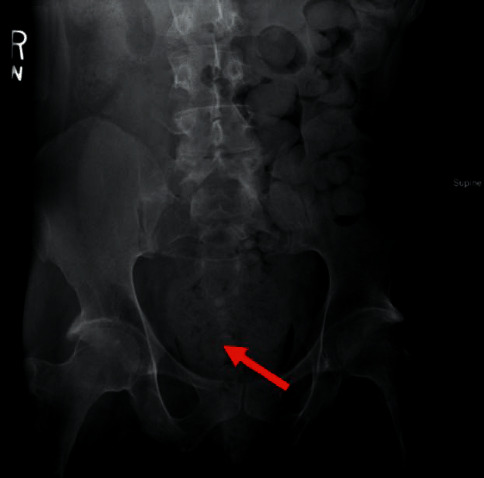
Abdominal X-ray radiography demonstrating fecaloma in a 34-year-old patient with intellectual and developmental disabilities and chronic constipation. The image shows a 10.2 cm fecaloma (red arrow) located in the rectum, with the presence of a high colonic stool burden proximally.

The most common complications arising from chronic constipation are hemorrhoids, anal fissures, and rectal bleeding. More serious complications include fecal incontinence (where overflow incontinence may confuse the diagnosis of chronic constipation), fecaloma, pelvic organ prolapse, fecal impaction, bowel obstruction necessitating surgery, and bowel perforation and stercoral peritonitis where extremely impacted feces can compress the colonic wall, causing an ischemic ulcer and subsequent perforation, culminating in stercoral peritonitis and sometimes death [25]. Fecaloma is a not uncommon complication of constipation, which is a stone that is formed by a high colonic stool burden and coprostasis. Patients may note a mass that protrudes from the anus and retracts with defecation [26] (Figure 1).

### 2.4. Treatment

The current literature does not provide a clear guideline on how to treat constipation in adults with IDD. Most of studies found in the literature address treatment of constipation in the pediatric age group [25]. The treatment of constipation in adult patients with IDD should mirror the management used for patients without IDD who have constipation [27].

Treatment of underlying conditions should be considered before empirically treating constipation. For instance, proper screening and treatment of hypothyroidism, which is a common comorbidity, is warranted [28]. Medication list should be carefully reviewed, trying to avoid medications that can cause constipation and finding alternative pharmacological and nonpharmacological means.

Patients with IDD who have behavioral issues and are on antipsychotic treatment causing constipation should be closely monitored. Treatment of behavioral issues should focus on resolving the distressing stimulus and behavioral therapy. Antipsychotics should be used as a last resort in circumstances where problematic behavior is refractory to therapy [29].

The initial treatment to relive the distressing symptoms of constipation may focus on increased dietary fiber intake and/or fluid intake depending on the underlying cause [6]. A crossover RCT showed that increased intake of whole fruits and vegetables significantly reduced fecal transit time by 14 h and increased the number of daily bowel movements by 0.4 and daily wet fecal weight by 118 g compared to 100% fruit and vegetable juices [30].

Agents known as “bulk laxatives” (Table 1) can be used to increase fiber intake and promote stooling. It should be noted that while some patients may benefit from a fiber-rich diet, many patients with more severe constipation may experience a worsening of symptoms with increased dietary fiber intake. Increased fluid will only improve constipation in select patients with underlying dehydration [31].

Patients sometimes fail therapy with increased dietary fiber intake and bulk-forming laxatives. At this point, surfactants or stool softeners can be started. Examples include docusate sodium and docusate calcium, which can be taken 1 to 2 times per day. These agents work by increasing water intake into the stool. Oftentimes, additional treatment is required. However, if further treatment is required, an osmotic agent should be regularly used and supplemented by a stimulant laxative as needed for “rescue” purposes. Osmotic agents result in the secretion of water into the intestinal lumen to promote loose stools. Examples of osmotic agents include polyethylene glycol-based solutions, magnesium citrate-based products, sodium phosphate-based products, and nonabsorbable carbohydrates. Stimulant laxatives stimulate intestinal motility and contraction. Examples of stimulant laxatives include bisacodyl and glycerin suppositories. Certain osmotic agents, such as polyethylene glycol, have more short-term and long-term efficacy when compared to stimulant laxatives [6].

Newer agents for the treatment of constipation include secretagogues and serotonin 5-HT4 receptor agonists. Secretagogues stimulate the net efflux of ions and water into the intestinal lumen and accelerate transit. Lubiprostone and linaclotide are examples of secretagogues. Serotonin 5-HT4 receptors are found on enteric neurons. Serotonin 5-HT4 receptor agonists allow the release of excitatory neurotransmitters, such as acetylcholine, to induce mucosal secretion. It is important to note that a trial of traditional approaches, including fiber supplementation, osmotic agents, and stimulant laxatives, are just as effective and safe compared with the newer agents [6].

Abdominal massage is a frequently used tool in the pediatric age group to manage constipation. However, it is no longer standard of care but may be a desirable therapy for this condition because it is inexpensive, noninvasive, and free of harmful side effects [32].

A randomized crossover study comparing abdominal massage (five times a week for 20 minutes) to the usual laxative regimen in adults with IDD living in group homes found no significant difference [33].

**Figure 2:**
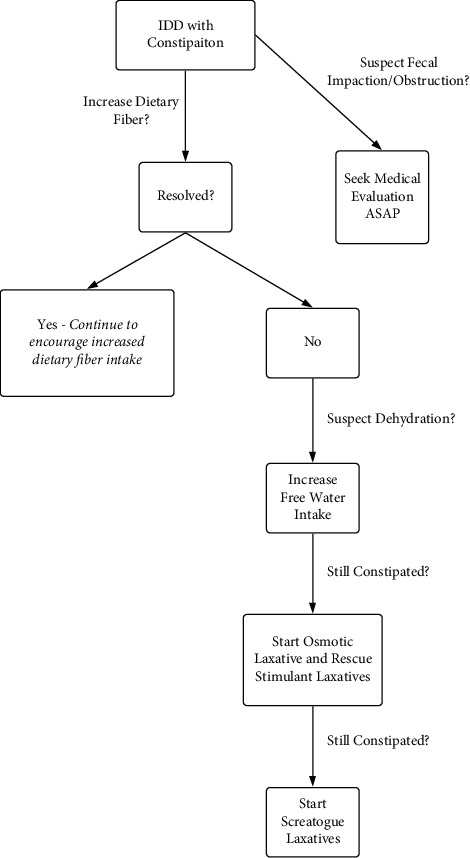
Treatment algorithm: a flow chart that helps PCPs in the step-by-step management of constipation in adults with IDD.

Patients with severe constipation and a large stool burden may experience fecal impaction and obstruction. If suspected, patients and their families should seek medical care in a timely manner (Figure 2). Patients with fecal impaction should have manual fragmentation and disimpaction [21]. Surgery is rarely required and should only be used as a last resort. It remains an option in patients with refractory constipation [21]. Total colonic resection and ileorectostomy should be considered in patients with constipation refractory to the medical therapies discussed above [22]. However, these procedures should not be used in patients with a defecatory disorder. Common complications after surgery include small bowel obstruction, diarrhea, and incontinence [21].

## 3. Conclusions

Constipation remains one of the major health problems in patients with IDD. There should be increased awareness of the risk of constipation in this patient population, due to the patient's inability to communicate their discomfort or needs. Chronic constipation and subsequent complications may lead to unnecessary anxiety, pain, discomfort, behavioral difficulties, and unnecessary complications in these already vulnerable patients. Encouraging patients with IDD to increase activity levels and improve nutrition are proactive ways to prevent constipation. Caregivers should also attempt to implement proper toileting routines, where healthcare providers should optimize medication management and avoid overuse of antipsychotics. In absence of a clear guideline of treatment of constipation in adults with IDD, treatment should be individualized and follow the same principle of treating constipation in adults without disabilities. With increased awareness of this issue, healthcare providers can improve health and wellbeing for patients with IDD.

## Figures and Tables

**Table 1:** Laxative choices.

Class	Class 2	Class 3
*Bulk-forming laxatives*		
Psyllium	12 to 72 hours	Impaction above strictures, excessive gas
Methylcellulose	12 to 72 hours	
Polycarbophil	24 to 48 hours	

*Surfactants*		
Docusate sodium	24 to 72 hours	Contact dermatitis has been reported
Docusate calcium	24 to 72 hours	

*Osmotic agents*		
Sorbitol	24 to 48 hours	Bloating, excessive gas
Polyethylene glycol	1 to 4 days	Nausea, bloating
Glycerin (suppository)	15 to 60 minutes	Local site irritation at the rectum
Magnesium sulfate	0.5 to 3 hours	Mag toxicity if used in excess, urgent defecation
Magnesium citrate	0.5 to 3 hours	The same as above

*Stimulants*		
Bisacodyl (oral)	6 to 10 hours	Gastric irritation
Senna	6 to 12 hours	Melanosis coli

Secretagogues		
Lubiprostone	24 to 48 hours	Nausea, diarrhea, and bloating
Linaclotide	12 to 24 hours	
Plecanatide	12 to 24 hours	
